# Intraoperative Imaging in Hepatopancreatobiliary Surgery

**DOI:** 10.3390/cancers15143694

**Published:** 2023-07-20

**Authors:** Tereza Husarova, William M. MacCuaig, Isabel S. Dennahy, Emma J. Sanderson, Barish H. Edil, Ajay Jain, Morgan M. Bonds, Molly W. McNally, Katerina Menclova, Jiri Pudil, Pavel Zaruba, Radek Pohnan, Christina E. Henson, William E. Grizzle, Lacey R. McNally

**Affiliations:** 1 Department of Surgery, University of Oklahoma Health Science Center, Oklahoma City, OK 73104, USA; 2 Department of Surgery, Military University Hospital Prague, 16902 Prague, Czech Republic; 3 Department of Radiation Oncology, University of Oklahoma Health Science Center, Oklahoma City, OK 73104, USA; 4 Department of Pathology, University of Alabama at Birmingham, Birmingham, AL 35294, USA

**Keywords:** intraoperative imaging, hepatopancreatobiliary surgery, targeted imaging, image-guided surgery

## Abstract

**Simple Summary:**

There is evidence that oncological radicality with complete tumor removal may improve the survival of patients undergoing surgery for hepatopancreatobiliary malignancies. However, the complexity of vital vascular structures close to or embedded within the pancreas and the liver may increase both the surgical difficulty and the risk of achieving a non-radical resection. Preoperative staging after neoadjuvant therapy is usually challenged by the inability of correctly used imaging methods to distinguish vital tumors from fibrosis. Additionally, the inability to define the exact tumor borders often transfers to the operating room as well. Recently, more research has focused on the development of novel intraoperative imaging modalities and targeted contrast agents to improve preoperative and intraoperative diagnostics. We review current advances made in preclinical research and discuss clinical possibilities and future perspectives, including the characteristics of the ideal contrast agent.

**Abstract:**

Hepatopancreatobiliary surgery belongs to one of the most complex fields of general surgery. An intricate and vital anatomy is accompanied by difficult distinctions of tumors from fibrosis and inflammation; the identification of precise tumor margins; or small, even disappearing, lesions on currently available imaging. The routine implementation of ultrasound use shifted the possibilities in the operating room, yet more precision is necessary to achieve negative resection margins. Modalities utilizing fluorescent-compatible dyes have proven their role in hepatopancreatobiliary surgery, although this is not yet a routine practice, as there are many limitations. Modalities, such as photoacoustic imaging or 3D holograms, are emerging but are mostly limited to preclinical settings. There is a need to identify and develop an ideal contrast agent capable of differentiating between malignant and benign tissue and to report on the prognostic benefits of implemented intraoperative imaging in order to navigate clinical translation. This review focuses on existing and developing imaging modalities for intraoperative use, tailored to the needs of hepatopancreatobiliary cancers. We will also cover the application of these imaging techniques to theranostics to achieve combined diagnostic and therapeutic potential.

## 1. Introduction

**Figure 1:**
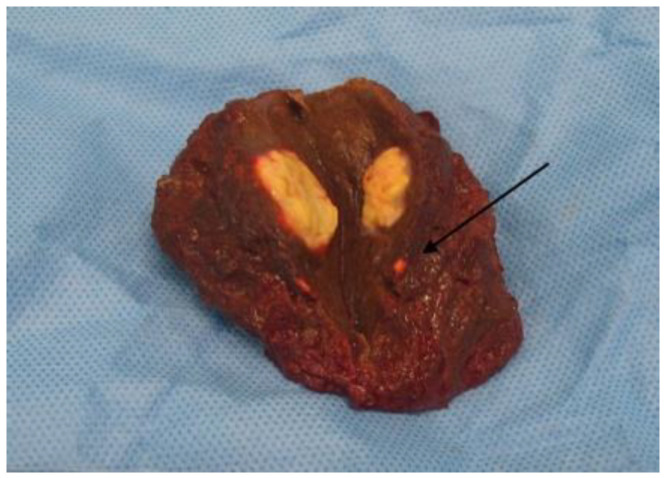
Satellite lesion (black arrow) of colorectal carcinoma liver metastasis with borderline visibility on ultrasound or CT. Original figure.

The current practice of perioperative imaging in hepatopancreatobiliary (HPB) surgery relies on a combination of imaging modalities including computed tomography (CT), magnetic resonance (MR), ultrasound (US), and positron emission tomography CT (PET/CT). Preoperative diagnostics have been improved by using organ-specific protocols [1,2,3,4], higher resolutions, complementary endoscopic imaging [5], and diagnostic biopsies. Even with these advances, the inability to differentiate peritumoral or chemotherapy-induced inflammation and fibrosis from the tumor itself persists; the detection of early stages of dissemination or determination of the extent of malignancy close to the dense vasculature of the upper gastrointestinal tract remains challenging. Often, small satellite lesions and microscopic margins are not evident with available imaging modalities, secondary to alterations in tissue composition due to other disease processes, examples being pancreatitis, steatosis of the liver, and cirrhosis (Figure 1). Intraoperatively, the surgeon’s only tools to navigate these difficulties are US, fresh frozen sectioning (FFS), and experience. FFS is the standard of care for intraoperative guidance, but there are numerous limitations to this method. These are mainly the length of the process, analysis limited to small amounts of tissues, analysis of small operative areas, and a notable discrepancy between FFS and definitive histopathology of up to 12.9% [6,7]. Importantly, if the FFS margins were positive, the tumor is not resected in-block. Obtaining additional margins in HPB surgery is often not possible, as further resection may require the resection of vital vasculatures such as the superior mesenteric artery in pancreas surgery, the portal triad, or hepatic venous structures, which would result in a too-small future liver remnant in the liver resection.

Perhaps advances made in the use of intraoperative US in HPB surgery depict the best potential for intraoperative imaging. Studies proving the added prognostic value of the intraoperative utilization of indocyanine green (ICG) with near-infrared (NIR) light sources are also emerging [8]. Currently, even the most complex surgeries are performed using mini-invasive approaches, where the concept of reliable and functional intraoperative NIR fluorescent imaging is even more appealing given the absence of tactile evaluation [9]. 

Owing to the limitations of current imaging and the established clinical benefit of molecular-image-guided surgery in neurosurgery [10] and gynecology [11], more research is aimed at the development of precise and accessible real-time imaging modalities in HPB surgery. This review considers the advances and perspectives of each imaging method, defines the characteristics of the ideal contrast agent, and describes the difficulties in clinical translation.

## 2. Methods

This is a narrative review synthesizing information from a literature search including the terms “hepatopancreatobiliary”, “intraoperative imaging”, and “novel imaging techniques”, as well as primary evidence from known examples.

## 3. Where Do We Stand? 

### 3.1. Ultrasound 

**Figure 2:**
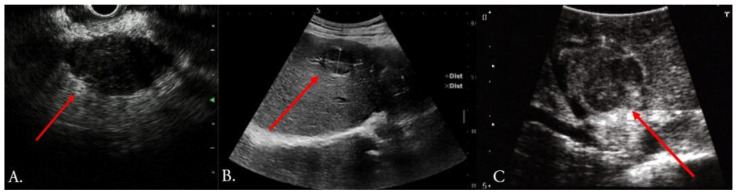
Commonly used ultrasound methods (red arrow indicates mass): (**A**) endoscopic ultrasound of pancreatic lesion, (**B**) preoperative transabdominal ultrasound of HCC liver lesion, (**C**) intraoperative ultrasound of HCC liver lesion close to the vasculature (identical lesion to image (**B**)). Original figure.

**Figure 3:**
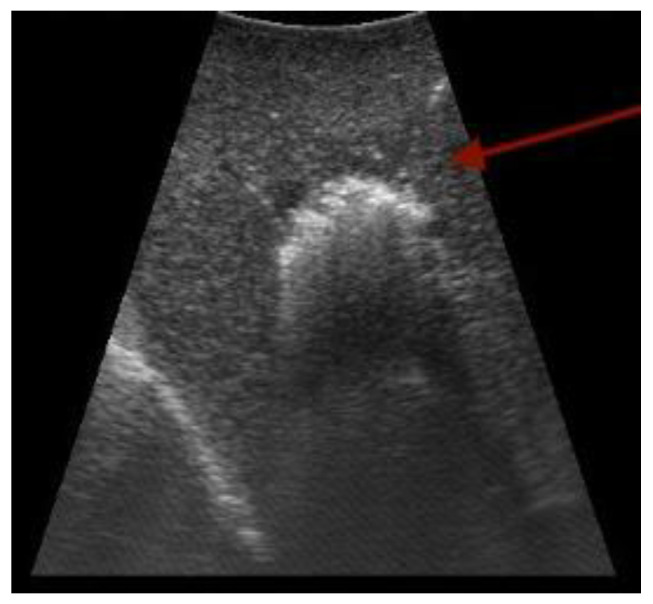
Ultrasound-guided radiofrequency ablation in the liver (arrow points toward the needle). Original figure.

US is used transabdominally and endoscopically (EUS); it is also the only routinely used intraoperative imaging modality in HPB surgery (IOUS) (Figure 2). Techniques such as contrast enhancement (CEUS), doppler mode, or elastography have also been implemented [12]. US became a standard of care in any surgical facility performing liver surgery in order to image complex and individually variable areas of liver anatomy and to improve tumor detection in real time; ablative liver therapies are not feasible without the use of IOUS [13] (Figure 3). The profound benefits and advances made with the use of IOUS in liver surgery are well documented [14,15,16]; the “radical but conservative” strategy of G. Torzilli et al. in performing more complex resections using IOUS led to a shift from major liver resections to more precise and complex parenchyma-sparing surgeries [17]. Even today, in the era of high-resolution MR and CT, IOUS, or more precisely, contrast-enhanced intraoperative ultrasound (CEIOUS), have superior qualities [16,18], and their accuracy has a fundamental effect on surgical strategies in liver surgery [19,20]. Nevertheless, the intra-operative use of US requires expertise, with a notable learning curve of 40 pancreatic and 50 liver cases [21]. Other potential problems include a lack of precise visibility in subcapsular areas and lesions less than 5 mm [22,23]. Image quality is limited in the setting of liver disease and in the area where ablative therapy was previously used. Also, there is currently an insufficient amount of data on the outcomes of US use in laparoscopic surgery [24].

### 3.2. Optical Imaging with Fluorescent Agents

A well-studied method in HPB surgery uses optical imaging with near-infrared fluorescence (NIR) agents [25,26,27]. Infrared light has a better tissue penetration, of up to 1 cm, compared with visible light, and the minimal autofluorescence of tissues in the NIR spectrum improves the target-to-background ratio (TBR) [28]. As multispectral cameras with a fusion of RGB and NIR imaging are widely available, imaging is fast and includes no damaging radiation. NIR imaging was initially used to determine cardiac output and hepatic function [29,30]. At present, its spectrum of use includes the assessment of tissue perfusion, ophthalmic angiography, sentinel lymph node evaluation, ureter visualization, and tumor mapping. 

The only FDA- and European Medicines Agency-approved fluorescent dyes are indocyanine green [31] and methylene blue (MB) [32]. Both dyes have been implemented in HPB surgery as perfusion agents. MB is often used as a visible stain as it is rapidly recognizable to the naked eye. However, with an excitation peak of around 700 nm, background tissue shows more autofluorescence in fluorescent images. The potential use of MB in HPB surgery is based on the ability to mark anatomic liver parenchymal resection margins [33] and detect bile leaks after liver resections [34]. 

The use of ICG is more widespread because it has an excitation peak of approximately 800 nm. This peak allows for superior visualization compared with MB because of the elimination of background autofluorescence, although it is difficult to detect with the eye alone, in contrast to MB [35]. Consensus guidelines for the use of fluorescence imaging in hepatobiliary surgery were published in 2021 by a group of Asia-Pacific experts [36] focusing on ICG use in liver and biliary surgery. ICG can be injected directly into the biliary system or intravenously, which could be considered a safer route. After intravenous application and binding to plasma proteins, ICG is taken up by hepatocytes and then fully eliminated via bile. These kinetics make it effective for imaging the extrahepatic biliary anatomy [37,38,39], a particularly beneficial trait in cases with anomalous or intricate biliary anatomies. Despite the notable benefits, ICG is unable to visualize the intrahepatic biliary tree because of limited tissue penetration or precisely identify common bile duct stones, probably because of the high concentrations of dye in bile. 

**Figure 4:**
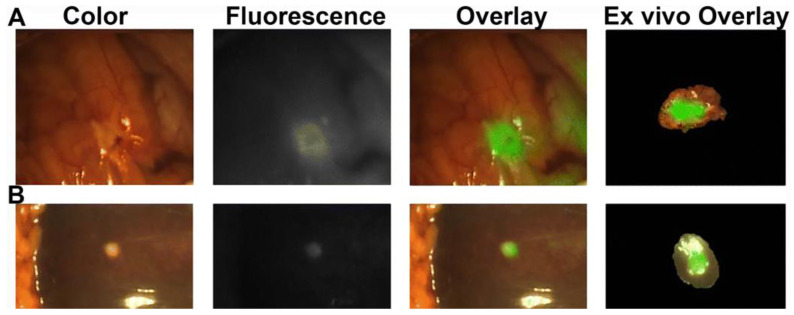
Visualization of a fluorescent anti-carcinoembryonic antigen–antibody in peritoneal (**A**) and liver (**B**) metastases of a pancreatic tumor, 48h post-intravenous injection [57]. Figure from *Ann. Surg. Oncol.* **2018**, *25*, 3350–3357 [57].

In tumor imaging, ICG has a sensitivity of up to 99% in identifying hepatocellular carcinoma (HCC) lesions [40], which has led to a more widespread use primarily in eastern countries, where the incidence of HCC is high. Yet, the average rate of false positivity is 10.5% [41]. Colorectal liver metastases (CRLM) tend to display a rim pattern of fluorescence; this may be due to the extensive central necrosis that is common in CRLM, or possibly, it may be due to distorted biliary extraction in immature hepatocytes surrounding the tumor tissue that, conversely, do not uptake the dye [42]. A newly published study on imaging superficial CRLM using ICG identified the ability to detect “disappearing lesions” after downstaging chemotherapy in 15 patients [43]. While the depth of the visualization is the main limit of ICG imaging, combining fluorescence and IOUS has proven to be superior to preoperative CT or IOUS alone in the detection of CRLMs ≤ 3 mm [23]. “Positive” and “negative” staining techniques have been described to help guide anatomic resection margins [44]. Apart from better intraoperative visualization and proof of concept data (Figure 4), studies of patient outcomes have emerged, showing the superiority of ICG-assisted liver surgeries for distinct indications [8,45]. 

The use of nonspecific dyes in pancreatic surgery has many limitations, and only a few reports exist. Healthy pancreases show a similar ICG uptake to tumor tissue, which creates ineffective tumor-to-background ratios (TBR) in carcinomas [46]. The way to potentially improve TBR with nonspecific dyes is using a second window technique (high-dose ICG injected intravenously 24 h prior to surgery). Given its enhanced permeability and retention effect (EPR), a TBR of 4.42 was achieved in 20 patients with malignant lesions enrolled in an open-label clinical trial [45,47]; three out of eight benign lesions were fluorescent as well.

Shirata et al. used intravenous ICG injected intraoperatively to image pancreatic ductal adenocarcinoma (PDAC), pancreatic NETs, and cystic neoplasms in 23 consecutive patients, proving the proposed hypothesis of visualization based on the hypovascularization (cystic lesions) or hypervascularization of lesions [48]. The reported TBR of the NETs was 1.99, with all the lesions successfully visualized intraoperatively (100% sensitivity). Cystic neoplasms showed lower fluorescent signals and a TBR of 0.54, with fair-to-poor visualization. The TBR of PDAC was of no statistical significance. These data are in line with other presented case reports; the COLPAN study reported 100% sensitivity in the laparoscopic ICG imaging of pancreatic NETs with a mean TBR of 7.7, peaking at 20 min after intravenous application [49]. Hutteman et al. reported no clear visualization of pancreatic carcinoma using intraoperative ICG, with a mean TBR of 1.22 ± 0.39 [46]. A recently published meta-analysis of ICG use in pancreatic surgery found six papers with a total of 64 lesions reported [50]; the overall sensitivity for all pancreatic lesions was 75% and a mean TBR of 1.22. The authors correctly pointed out the unknown effect of neoadjuvant therapy on visualization and the unknown effects of ICG use on recurrence-free and overall survival in pancreatic surgery.

While recommendations on ICG dosage and the timing of administration in liver and biliary surgery exist [36], there is a large inter-institutional discrepancy between dosages, ranging from a bolus of 2.5 mg to 5 mg/kg, and the timing of ICG administration in pancreatic tumor imaging [45,46]. Perhaps one of the main challenges is the fact that, while liver metastasis and the primary tumor site may benefit from the second window technique, peritoneal metastases are likely to be missed at the time of exploration and could require intraoperative ICG applications [51]. In contrast, the administration of ICG intraoperatively leads to unavoidable background fluorescence in the liver and biliary tract. As the current papers suggest, different pancreatic neoplasms will probably require different ICG administration timing. The timing of administration is currently being studied in a Japanese trial: jRCT1051180076.

Pancreatic surgery is burdened with high rates of R1 resections in up to 80% of cases [52,53] and difficulty in staging patients after neoadjuvant therapy, especially in evaluating the degree of treatment response within the tumor. The high rate of R1 resections is partially influenced by vascular invasion and a previous lack of widespread adoption of standardized pathologist protocols [54]. R0 resection is, however, an independent prognostic factor of survival after pancreatic resection [52], therefore, the ability to precisely image pancreatic tumors in the operating room would be an important milestone in pancreatic surgery. The era of minimally invasive surgery, where tactile feedback is omitted, calls for even more precise intraoperative imaging. New methods emerge with the aim of improving fluorescent imaging, e.g., the second near-infrared wavelength window NIR-II (1000–1700 nm), which suggests a higher sensitivity and detection of up to 8mm deep [55]. More studies evaluating the impact of fluorescence on intraoperative decision making are needed. Reporting was suggested by Lauwerends et al. [56].

### 3.3. Optoacoustic-Based Imaging

Optoacoustic (OA), or photoacoustic imaging, is another emerging method with significant potential. The principle of “light-in, sound-out” is utilized [58]; the absorption of near-infrared light generates acoustic waves via the thermoelastic expansion of tissues that are detected using a computerized transducer. In biological tissues, sound scatters 1000 times slower than light, circumventing the resolution/imaging depth tradeoff that hinders the application of optical imaging [59]. In addition, as biological tissue is largely transparent to near-infrared light, imaging depths of approximately 5 cm are achievable with no sacrifices in resolution [60,61]. While optical imaging agents, i.e., ICG, methylene blue, and IR-800-CW dyes, are detectable using both optical imaging and optoacoustic imaging, these agents are optimized to generate the largest fluorescence signal and represent suboptimal optoacoustic agents. Optical imaging with agents such as ICG requires a path length double of that of optoacoustic imaging while also utilizing higher-energy light, which is more susceptible to photon absorption and scatter in biological tissue. This inherent disadvantage of fluorescence restricts imaging to an imaging depth of <6 mm to maintain suitable resolution [62]. However, optoacoustic signals have been detected as several centimeters. Optoacoustic imaging is often conducted in individual perpendicular planes, which may be combined following acquisition to establish a 3D tomographic image. This provides an advantage in HPB surgical imaging, as it allows for the visualization of tumors within the liver and pancreatic parenchyma, as they are often deeper than 6 mm from the anterior surface, and molecular imaging agents have been detected within the liver, i.e., high hemoglobin content, in preclinical models [63].

**Figure 5:**
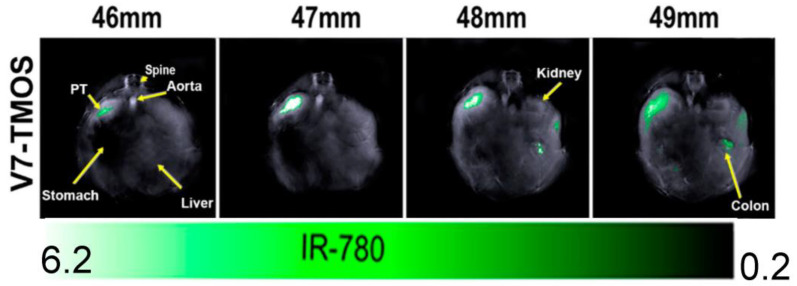
Visualization of optoacoustic nanoparticle accumulation in a pancreatic tumor of a xenograft murine model. Each image represents a different tomographic slide in the animal. Figure adapted from *ACS Appl. Mater. Interfaces* **2021**, *13*, 49614–49630 [71].

**Figure 6:**
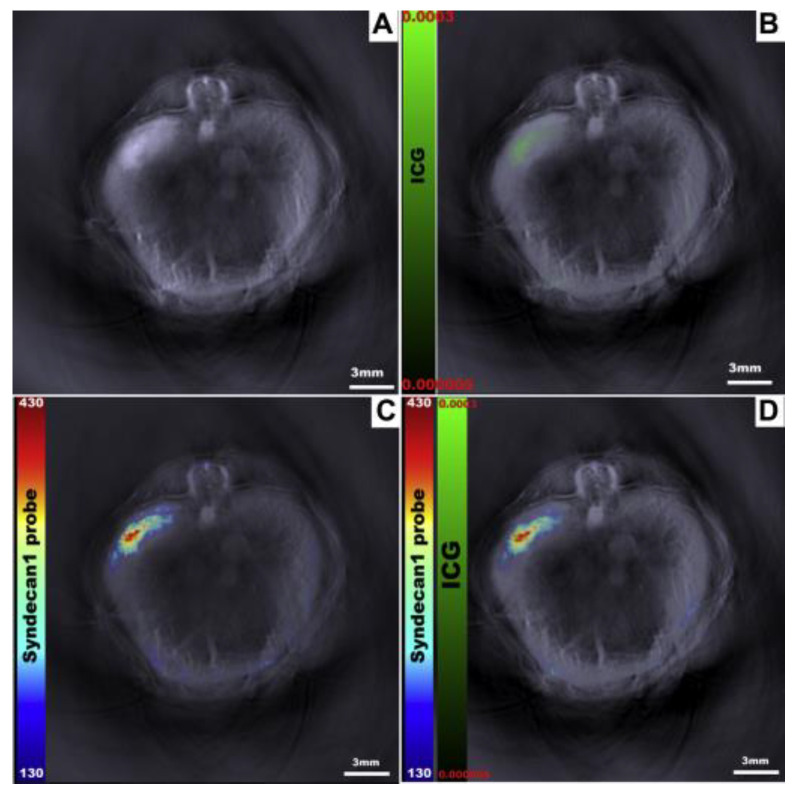
Visualization of multiple optoacoustic contrast agents using single wavelength 900nm (**A**) and with multiple wavelengths separately (ICG (**B**), Syndecan1 probe (**C**)) and simultaneously (**D**) following spectral unmixing using an in vivo murine model. Figure from *J. Surg. Res.* **2015**, *193*, 246–254 [75].

Both endogenous and exogenous contrast agents can be detected with optoacoustic imaging. While endogenous contrast agents eliminate the problems of utilizing dyes in tissues, they are usually weak reporters with non-unique spectra [64]. However, there is a current lack of contrast agents that are developed and optimized for optoacoustic imaging [62,63,64,65,66]. The unique optical properties, e.g., optical absorption as a function of wavelength, of different contrast agents lead to the ability of “unmixing” images to determine the location and concentration of each unique agent simultaneously. This unmixing capability in the context of optoacoustic imaging is called multispectral optoacoustic tomography (MSOT). Specifically, multiwavelength illumination is used to identify the absorption and emission spectra for every contrast agent in an area to differentiate between background and individual contrast agent signals in a murine model (Figure 5). This capability could be utilized to provide information about a tumor’s molecular features and/or structure’s metabolism within tissues [67,68,69,70,71,72,73]. For example, a response to oxyhemoglobin may outline an artery in the vicinity of angiogenesis as a symptom of tumor progression. The development of OA agents can significantly improve the capabilities of in vivo imaging, such as identifying deep tumors following the administration of an OA agent in a murine model (Figure 6). This is a major advantage when aiming to distinguish tumors from peri-tumoral fibrosis, necrosis, and inflammation. For example, benign and malignant gallbladder polyps were shown to have different OA signal intensities [74]. 

The visualization of molecules within 200 uM blood vessels can be achieved without the artifacts associated with vascularly dense tissues or the vessels themselves with NIR [75,76,77]. In contrast, ICG loses fluorescence intensity after binding to proteins in blood vessels when visualized with NIR [78], therefore limiting use in highly vascular tissues, which is often the case in HPB surgery. Furthermore, the ICG signal may be hindered further by blood pooling within the surgical field secondary to blood loss, which is not uncommon in hepatobiliary surgery [79]. Of importance, hemoglobin is one of the few strong endogenous contrast agents that allow for the identification of microvascular changes and tissue oxygenation using MSOT [58,80], and it has been successfully used to monitor tumor responses to antiangiogenic agents in mouse models [81]. Exogenous contrast agents used for OA imaging in preclinical mouse studies include organic dyes, such as ICG, and nanoparticles, such as gold, silver, tungsten, iron oxide, and carbon nanotubes [64,70,71]. OAI showed a 3.7 TBR in the resected specimens of patients with PDAC when targeted with cetuximab-IRDye800, a NIR fluorescent agent that binds to the epidermal growth factor receptor [82]. This study, as well as several others [83], tried to combine fluorescence with OAI in a multimodal approach; fluorescence had a sensitivity of 96.1%, a positive margin was identified, and the separate differentiation of chronic pancreatitis from PDAC was possible. However, the restricted depth of imaging and relatively high level of false positive lesions remains the main limitation of ICG with OAI. 

Besides frequent subcutaneously grafted tumor models, MSOT has been successfully used in orthotopic pancreatic cancer mouse models and created 3D images [61,75,83], which has driven further interest in this modality. However, while ICG is readily available and a contrast agent known to most surgeons, more contrast agents that are specifically designed for optoacoustic imaging are needed for further clinical translation.

### 3.4. Photodynamic Imaging 

The concept of photodynamic diagnosis, or photodynamic imaging (PDD), utilizes the application of photosensitizers that accumulate in targeted tissues that can then be imaged upon excitation using specific infrared (IR) wavelengths. The photodynamic effects of ICG and similar molecules are expected upon excitation with an infrared laser (805 nm) during the surgical procedure.

Most of the data on photodynamic imaging are in preclinical proof-of-concept studies in small case series in various proposed areas of HPB surgery, notably in the successful imaging of HCC using ALA [84,85] and the determination of tumor margins for cholangiocarcinoma in a mouse model [86]. Another study tested laparoscopic photodynamic imaging to detect carcinomatosis in a staging laparoscopy for pancreatic cancer. Staging laparoscopies are a common practice for high-risk patients with HPB malignancy because of a lack of ability in preoperative imaging to precisely determine the early stages of peritoneal dissemination; however, this is currently solely dependent on the surgeon’s experience. Indicating an area that needs more improvement, the study reported an increased rate of laparoscopic detection of peritoneal dissemination with fluorescence and even higher rates with spectrophotometry compared with white light in mouse models. Perhaps the greatest limitation of PDD in laparoscopy, especially for ALA, which has emission peaks in the spectrum of red-to-near-infrared light, is the low concentrations of fluorophores not limited to superficial layers. The study used spectrophotometry to overcome this limitation. The rate of ex vivo detection in human specimens using spectrophotometry was 63% [87]. New photosensitizing agents and nanocarriers are being tested [88,89]. There is also an interest in finding adjuvant agents to accelerate protoporphyrin accumulation in tumors, e.g., the currently active clinical trial NCT03467789, which is evaluating the effect of vitamin D.

PDD possesses potential characteristics for real-time imaging, and the concept of combining PDD and photodynamic therapy (PDT) to monitor tumor responses to PDT or treat resection margins is perhaps appealing. While studies on PDT alone are ongoing in HPB—e.g., the current clinical trial NCT03033225 for the treatment of unresectable pancreatic cancer using PDT [90]—the concept of combining PDD and PDT is not documented to the best of our knowledge. More studies are needed to address the real potential of photodynamic imaging and/or therapy in clinical settings.

### 3.5. Intraoperative 3D Imaging 

Creating a three-dimensional image is a natural next step in today’s digital era [91]. Three-dimensional modeling and its derived volumetric calculations are not uncommon in liver and biliary surgery. Data proving more precise preoperative planning in liver resection for HCC in 3D vs. 2D were recently published [92]. The idea of supporting intraoperative orientation led to the creation of 3D holograms, or 3D modeling, which faced the challenge of changing organ shapes during intraoperative manipulation. Today, “last-minute simulation” is mostly reported rather than intraoperative navigation, necessitating more research [93,94].

## 4. Characteristics of Ideal Tracer for Molecular Imaging

In nuclear medicine, the clinical value of labeled tracers used to image and monitor the metabolic activity of targeted tissues has been well documented [95,96]. Similar tracer characteristics are required for intraoperative imaging, e.g., pharmacokinetics, costs, and safety. While the fast elimination of contrast agents in nuclear medicine is desired, intraoperative imaging may favor extending the imaging window throughout the resection phase of surgery or the possibility of the repeated use of contrast agents. Creating a detectable signal with low amounts of contrast agents and a high TBR, including differentiation between tumors and surrounding fibrosis and inflammation, should be the main goals when developing probes for intraoperative imaging. Of note, MSOT can detect collagen as a measure of fibrosis.

While the passive targeting of nonspecific dyes (e.g., fluorescent imaging HCC based on EPR) is possible, it requires higher concentrations of contrast agents and generally has a lower labeling efficiency [97], as discussed previously. Therefore, active targeting seems to be the ideal design, perhaps even reaching microdosing levels. Most targeting contrast agents consist of a targeting component and a signaling component [98], although a dye that would be tissue-specific is an appealing concept. The strong affinity of contrast agents for target tissues and their fast elimination from blood and off-targeted tissues ultimately create a high TBR. 

Antibodies are one of the most widely tested targeting agents because of their natural targeting ability and a range of molecules that are already FDA-approved [99]. Clinical translation has been, however, hindered by several challenges (Table 1). Antibodies have a fairly large molecular size (150 kDa), which limits penetration within solid tumors that typically possess dense stroma, e.g., stroma in pancreatic cancer form 60–90% of the tissue [100]. Solid tumors also have various regions of hypoxia and acidity, which may affect the binding capacity of antibodies [101]. Furthermore, a notable intertumoral and interpatient variability in the overexpression of targeted receptors exists [102]. The high costs and administration several days before surgery must also be considered. Both anti-VEGFR (bevacizumab) [103] and anti-carcinoembryonic antigen (CEA) [57,104], conjugated to a fluorescent dye, showed suboptimal signal-to-background ratios, ranging from 1.4 to 2.1 when tested on patients with pancreatic and colorectal cancer. This prompted the early termination of the NCT 02743975 trial using bevacizumab. A study using anti-CEA antibodies for the detection of colorectal and pancreatic cancer liver metastasis in human patients showed a mean TBR of 1.7, with two false positives out of nineteen lesions [105]. Interestingly, the false-positive lesions had no CEA expression, indicating parallel mechanisms of antibody retention or fluorescence signal.

Given that an antibody’s size may be a limiting factor, antibody fragments with similarly low immunogenic potential have emerged in preclinical testing. The variable kinetics of molecules of different sizes, binding affinities, and half-life influence further diffusivity, EPR effects, and the clearance and homogeneity of tracer distributions within tumors [106]. Antibodies generally compensate for slow extravasation with prolonged circulation due to minimal renal clearance (thus requiring administration several days ahead of surgery). While decreasing the size of a particle leads to increased vascular permeability (allowing for even same-day imaging [107]), this also leads to faster renal clearance, rapidly lowering the intravascular concentration needed for diffusion into tumorous tissue. A recent in vivo study on mouse models, including pancreatic ductal adenocarcinoma models, showed that, while imaging is faster using nanobodies, peak fluorescence is lower, contrary to the same antibody [108]. Nanobodies have high specificity, low background retention, and more homogenous tissue penetration [109]; furthermore, they are stable, making them ideal for further modifications and labeling, which leads to an unlimited range of possible particles to be developed and tested. A spectrum of nanoparticles, peptides, and coated particles was tested in vitro and in animal models [98,110] (Table 2 and Table 3); the importance of the timing of administration and dosage was also demonstrated in preclinical settings [107], but a clinical translation is yet to be started (an overview of the advantages and disadvantages of ligands based on their sizes is in Table 4).

Another important consideration is whether a tracer has an extracellular or intracellular target; catalyzes an enzymatic reaction; or mediates a chemical transformation [98]. Solid tumors have significantly lower pH because of specific cancer cell metabolisms; thus, targeting acidic pH is another appealing option [111]. The mechanism is interesting since the effectivity of many other tracers might be oppositely or negatively influenced by acidity.

**Table 1:** An overview of targeted agents used in clinical trials.

Molecule	Imaging Technique	Phase	Target	Imaged Cancer	Administration-to-Imaging Time	Clinical Trial Number, Reference
SGM-101	NIR	I.	CEA	Colorectal and pancreatic cancer liver metastases	4 days	[105]
Anti-GPC3-IRDye800CW	NIR II.	I.	GPC3	HCC	Not specified	NCT05047510
SGM-101	NIR	I.	CEA	PDAC	48 h	[104]
Penitumumab-IRDye800	NIR	I./II.	EGFR	PDAC	58 h	NCT03384238
LUM015	LUM Imaging system	I./II.	Cathepsin proteases	Pancreatic cancer, colorectal cancer, esophageal cancer	1 h prior to pancreatic surgery; 2–6 h prior to colorectal surgery	NCT02584244
Bevacizumab-800CW	NIR	I.	VEGFR-A	PDAC	72 h	NCT02743975 [103]

**Table 2:** Comparison of different types of targeting agents.

Type	Small Molecule	Peptide	Aptamer	Monoclonal Antibody	Protein Fragment (Diabody)	Nanoparticle	Microbubble
Size	<0.5 kDa	0.5–2 kDa	5–15 kDA	150 kDa	55 kDa	10–100 nm	1–4 µm
Example	IR-800 CW dye with P47	Cyclic RGD peptide	TLS11a	Anti-Sp17-ICG-Der-02	[^18^F]SFB	Mesoporous silica nanoparticle	VEGFR-1
Advantage	Easily escapes vasculature	Easily modified; superior selectivity	Inexpensive production; high diversity	High affinity and specificity	Superior tumor penetration; high tumor-to-blood ratio	Effective delivery of signaling and therapeutic payload	Good with safety; wide availability of contrast-mode ultrasound scanners
Disadvantage	Costly development; limited size for the signaling component	Rapid degradation	Low in vivo stability; poor membrane passage	Slow clearance; restricted in passing biological barriers	Accumulation in the kidneys	Difficult extraversion because of size	Imaging limited to molecule targeted; differentially expressed on tumor vasculature
References	[112]	[113]	[114]	[115]	[116]	[117]	[118]

**Table 3:** Pancreatic-cancer-targeting agents in preclinical studies.

Molecule	Imaging Technique	Target	Administration to Imaging Time	Reference
6G5j-IR700DX	Fluorescence	CEA	24 h	[119]
Anti-MUC1 antibody conjugated with DyLight 650	Fluorescence	MUC1	24 h	[120]
cRGD-ZW800–1	NIR	Integrins	4 h	[121]
ssSM3E/800CW	NIR	CEA	24 h	[122]

**Table 4:** Liver-cancer-targeting agents in preclinical studies.

Molecule	Imaging Technique	Target	Administration to Imaging Time	Reference
FeSe2−PEG−peptide	PAI + MRI	GPC3 (HCC)	12 h	[123]
Den-Apt1	NIR+ MRI	Endoglin (HCC)	2 and 24 h	[124]
ACPP-Cy5	NIR	Activated by MMP-2 and MMP-9 (CRLM)	3 and 6 h	[125]
Gd@DOTA&IRDye800-SP94	NIR	HCC (Unknown target)	4 h	[126]
Zr-Df-YY146-ZW800	NIR + PET	CD146 (HCC)	4, 48, 120 h	[127]
huCC49-IR800	NIR	TAG-72 (CRLM)	48 h	[128]
Anti-Sp17-ICG-Der-02	NIR	Sp17 (HCC)	1, 2, 4, 6 h; 1, 2, 3, 7 days	[115]
ICG/MSNs-RGD	NIR	αvβ3 receptor (HCC)	10min, 24, 48, 72, 96, and 120 h	[129]
IRDye800CW-SAHA	NIR	Histone deacetylase, HDACs (HCC)	2, 4, 6, 12, 24, 48 h	[130]
Anti-CEA-DyLight650	NIR	CEA (CRLM)	24, 48, 72, 96 h	[131]
IRDye 800CW (IR800)-labeled P47	NIR	HCC	24 h	[112]
AF750-labeled AP613-1	NIR	GPC3 (HCC)	2 h	[132]
FAM-labeled RS peptide	NIR	HCC, CCC	2 h	[133]
ICG/Pt@PDA-CXCR4 (referred to as IPP-c)	PAI + NIR	CXCR4 (HCC)	5 and 24 h	[134]
AFP-antibody-modified magnetic liposome (nanoprobe)	NIR + MR	AFP	1, 6, 12, and 24 h	[135]
Fluorophore-conjugated IGF-1R antibody	Fluorescence	IGF-1R (CRC liver metastasis)	24 h	[136]
F12+-ANP-Gal	NIR	H2S activable (HCC HepG2)	12 h	[137]

## 5. Conclusions and Future Directions 

Significant progress in the treatment of HPB malignancies has been made over the last few decades, including advanced techniques for liver resections, improved systemic therapy, and perioperative care [138,139,140]. Complex liver resections, with the possibility of resecting up to 70% of the parenchyma, allow more patients to be considered for curative resections, and use a combination of resections and ablative techniques. Neither would be possible without the implementation and improvement of intraoperative imaging. Conversely, given that more patients on systemic therapy become eligible for surgical resection, preoperative staging and intraoperative orientation have become more grueling. Additionally, the pressure to perform more mini-invasive surgeries, including HPB surgeries, is palpable in Western countries. While in open surgeries, the surgeon’s tactile sensation is one of the main guiding instruments; its absence in laparoscopic surgery, and even more so in robotic surgery, can hinder the orientation. Lastly, preoperative preparation is crucial for the guidance of the surgery, but delays between staging and surgery are sometimes unavoidable, necessitating further reevaluation in the operating room.

All of the above requires further research and development in intraoperative imaging. Preclinical data show endless amounts of possibilities, but the superiority of one contrast agent over the others is yet to be demonstrated. Combinations of several modalities have been tested. From a surgeon’s perspective, intraoperative imaging needs to be precise and easily implemented and evaluated. The concept of the same-day administration of a contrast agent is appealing in the era of ERAS and fast-track surgeries. Importantly, the costs will have an important effect on the broad implementation of any new techniques. Multipurpose contrast agents aimed at preoperative, intraoperative, and possibly therapeutic use are not solely focused on qualities sought in the operating room, but the possibility of the broad clinical implementation of a drug is undeniably more appealing for development. The strenuous process of clinical translation should not discourage the further drive to improve intraoperative imaging given its potential and possible broad implementation. Connecting preclinical research with clinicians is critical in order to maximize the potential of research and resources. Vice versa, the surgical community needs to be open to helping facilitate the clinical translation of preclinical research. 

## References

[1] Fukukura Y., Kumagae Y., Fujisaki Y., Yamagishi R., Nakamura S., Kamizono J., Nakajo M., Kamimura K., Nagano H., Takumi K. (2021). Adding Delayed Phase Images to Dual-Phase Contrast-Enhanced CT Increases Sensitivity for Small Pancreatic Ductal Adenocarcinoma. AJR Am. J. Roentgenol..

[2] Donato H., Franca M., Candelaria I., Caseiro-Alves F. (2017). Liver MRI: From basic protocol to advanced techniques. Eur. J. Radiol..

[3] Choi J.Y., Lee J.M., Sirlin C.B. (2014). CT and MR imaging diagnosis and staging of hepatocellular carcinoma: Part I. Development, growth, and spread: Key pathologic and imaging aspects. Radiology.

[4] Choi J.Y., Lee J.M., Sirlin C.B. (2014). CT and MR imaging diagnosis and staging of hepatocellular carcinoma: Part II. Extracellular agents, hepatobiliary agents, and ancillary imaging features. Radiology.

[5] Gonzalo-Marin J., Vila J.J., Perez-Miranda M. (2014). Role of endoscopic ultrasound in the diagnosis of pancreatic cancer. World J. Gastrointest. Oncol..

[6] Voskuil F.J., Vonk J., van der Vegt B., Kruijff S., Ntziachristos V., van der Zaag P.J., Witjes M.J.H., van Dam G.M. (2022). Intraoperative imaging in pathology-assisted surgery. Nat. Biomed. Eng..

[7] Nelson D.W., Blanchard T.H., Causey M.W., Homann J.F., Brown T.A. (2013). Examining the accuracy and clinical usefulness of intraoperative frozen section analysis in the management of pancreatic lesions. Am. J. Surg..

[8] Liu F.S., Wang H.T., Ma W.J., Li J.H., Liu Y.Y., Tang S.L., Li K., Jiang P., Yang Z.Y., He Y.M. (2023). Short- and Long-Term Outcomes of Indocyanine Green Fluorescence Navigation-Versus Conventional-Laparoscopic Hepatectomy for Hepatocellular Carcinoma: A Propensity Score-Matched, Retrospective, Cohort Study. Ann. Surg. Oncol..

[9] Qin R., Kendrick M.L., Wolfgang C.L., Edil B.H., Palanivelu C., Parks R.W., Yang Y., He J., Zhang T., Mou Y. (2020). International expert consensus on laparoscopic pancreaticoduodenectomy. Hepatobiliary Surg. Nutr..

[10] Stummer W., Pichlmeier U., Meinel T., Wiestler O.D., Zanella F., Reulen H.J., Group A.L.-G.S. (2006). Fluorescence-guided surgery with 5-aminolevulinic acid for resection of malignant glioma: A randomised controlled multicentre phase III trial. Lancet Oncol..

[11] van Dam G.M., Themelis G., Crane L.M., Harlaar N.J., Pleijhuis R.G., Kelder W., Sarantopoulos A., de Jong J.S., Arts H.J., van der Zee A.G. (2011). Intraoperative tumor-specific fluorescence imaging in ovarian cancer by folate receptor-alpha targeting: First in-human results. Nat. Med..

[12] Bartos A., Iancu I., Ciobanu L., Badea R., Sparchez Z., Bartos D.M. (2021). Intraoperative ultrasound in liver and pancreatic surgery. Med. Ultrason..

[13] Lee J.Y., Kim Y.H., Roh Y.H., Roh K.B., Kim K.W., Kang S.H., Baek Y.H., Lee S.W., Han S.Y., Kwon H.J. (2016). Intraoperative radiofrequency ablation for hepatocellular carcinoma in 112 patients with cirrhosis: A surgeon’s view. Ann. Surg. Treat. Res..

[14] Torzilli G., Leoni P., Gendarini A., Calliada F., Olivari N., Makuuchi M. (2002). Ultrasound-guided liver resections for hepatocellular carcinoma. Hepatogastroenterology.

[15] Sietses C., Meijerink M.R., Meijer S., van den Tol M.P. (2010). The impact of intraoperative ultrasonography on the surgical treatment of patients with colorectal liver metastases. Surg. Endosc..

[16] Hoch G., Croise-Laurent V., Germain A., Brunaud L., Bresler L., Ayav A. (2015). Is intraoperative ultrasound still useful for the detection of colorectal cancer liver metastases?. HPB.

[17] Torzilli G., Montorsi M., Donadon M., Palmisano A., Del Fabbro D., Gambetti A., Olivari N., Makuuchi M. (2005). “Radical but conservative” is the main goal for ultrasonography-guided liver resection: Prospective validation of this approach. J. Am. Coll. Surg..

[18] Sahani D.V., Kalva S.P., Tanabe K.K., Hayat S.M., O’Neill M.J., Halpern E.F., Saini S., Mueller P.R. (2004). Intraoperative US in patients undergoing surgery for liver neoplasms: Comparison with MR imaging. Radiology.

[19] Torzilli G., Del Fabbro D., Palmisano A., Donadon M., Bianchi P., Roncalli M., Balzarini L., Montorsi M. (2005). Contrast-enhanced intraoperative ultrasonography during hepatectomies for colorectal cancer liver metastases. J. Gastrointest. Surg..

[20] Shah A.J., Callaway M., Thomas M.G., Finch-Jones M.D. (2010). Contrast-enhanced intraoperative ultrasound improves detection of liver metastases during surgery for primary colorectal cancer. HPB.

[21] Parks K.R., Hagopian E.J., Hagopian E.J., Machi J. (2014). Introduction: The Importance of Ultrasound in a Surgical Practice. Abdominal Ultrasound for Surgeons.

[22] Claudon M., Dietrich C.F., Choi B.I., Cosgrove D.O., Kudo M., Nolsoe C.P., Piscaglia F., Wilson S.R., Barr R.G., Chammas M.C. (2013). Guidelines and good clinical practice recommendations for contrast enhanced ultrasound (CEUS) in the liver--update 2012: A WFUMB-EFSUMB initiative in cooperation with representatives of AFSUMB, AIUM, ASUM, FLAUS and ICUS. Ultraschall Med..

[23] Peloso A., Franchi E., Canepa M.C., Barbieri L., Briani L., Ferrario J., Bianco C., Quaretti P., Brugnatelli S., Dionigi P. (2013). Combined use of intraoperative ultrasound and indocyanine green fluorescence imaging to detect liver metastases from colorectal cancer. HPB.

[24] van der Steen K., Bosscha K., Lips D.J. (2021). The value of laparoscopic intraoperative ultrasound of the liver by the surgeon. Ann. Laparosc. Endosc. Surg..

[25] Ishizawa T., Bandai Y., Ijichi M., Kaneko J., Hasegawa K., Kokudo N. (2010). Fluorescent cholangiography illuminating the biliary tree during laparoscopic cholecystectomy. Br. J. Surg..

[26] Ashitate Y., Stockdale A., Choi H.S., Laurence R.G., Frangioni J.V. (2012). Real-time simultaneous near-infrared fluorescence imaging of bile duct and arterial anatomy. J. Surg. Res..

[27] Aoki T., Murakami M., Yasuda D., Shimizu Y., Kusano T., Matsuda K., Niiya T., Kato H., Murai N., Otsuka K. (2010). Intraoperative fluorescent imaging using indocyanine green for liver mapping and cholangiography. J. Hepatobiliary Pancreat. Sci..

[28] van Manen L., Handgraaf H.J.M., Diana M., Dijkstra J., Ishizawa T., Vahrmeijer A.L., Mieog J.S.D. (2018). A practical guide for the use of indocyanine green and methylene blue in fluorescence-guided abdominal surgery. J. Surg. Oncol..

[29] Detter C., Wipper S., Russ D., Iffland A., Burdorf L., Thein E., Wegscheider K., Reichenspurner H., Reichart B. (2007). Fluorescent cardiac imaging: A novel intraoperative method for quantitative assessment of myocardial perfusion during graded coronary artery stenosis. Circulation.

[30] Dorshow R.B., Bugaj J.E., Burleigh B.D., Duncan J.R., Johnson M.A., Jones W.B. (1998). Noninvasive fluorescence detection of hepatic and renal function. J. Biomed. Opt..

[31] Alexandrov L.B., Nik-Zainal S., Wedge D.C., Aparicio S.A., Behjati S., Biankin A.V., Bignell G.R., Bolli N., Borg A., Borresen-Dale A.L. (2013). Signatures of mutational processes in human cancer. Nature.

[32] Debie P., Hernot S. (2019). Emerging Fluorescent Molecular Tracers to Guide Intra-Operative Surgical Decision-Making. Front. Pharmacol..

[33] Cai S., Yang S., Lv W., Geng C., Gu W., Duan W., Wang W., Huang Z., Dong J. (2015). Sustained methylene blue staining to guide anatomic hepatectomy for hepatocellular carcinoma: Initial experience and technical details. Surgery.

[34] Tuysuz U., Aktas H., Bati I.B., Emiroglu R. (2021). The role of Intraoperative cholangiography (IOC) and methylene blue tests in reducing bile leakage after living donor hepatectomy. Asian J. Surg..

[35] Van Keulen S., Hom M., White H., Rosenthal E.L., Baik F.M. (2023). The Evolution of Fluorescence-Guided Surgery. Mol. Imaging Biol..

[36] Wang X., Teh C.S.C., Ishizawa T., Aoki T., Cavallucci D., Lee S.Y., Panganiban K.M., Perini M.V., Shah S.R., Wang H. (2021). Consensus Guidelines for the Use of Fluorescence Imaging in Hepatobiliary Surgery. Ann. Surg..

[37] Ishizawa T., Bandai Y., Kokudo N. (2009). Fluorescent cholangiography using indocyanine green for laparoscopic cholecystectomy: An initial experience. Arch. Surg..

[38] Pesce A., Piccolo G., La Greca G., Puleo S. (2015). Utility of fluorescent cholangiography during laparoscopic cholecystectomy: A systematic review. World J. Gastroenterol..

[39] Gene Skrabec C., Pardo Aranda F., Espin F., Cremades M., Navines J., Zarate A., Cugat E. (2020). Fluorescent cholangiography with direct injection of indocyanine green (ICG) into the gallbladder: A safety method to outline biliary anatomy. Langenbecks Arch. Surg..

[40] Ishizawa T., Masuda K., Urano Y., Kawaguchi Y., Satou S., Kaneko J., Hasegawa K., Shibahara J., Fukayama M., Tsuji S. (2014). Mechanistic background and clinical applications of indocyanine green fluorescence imaging of hepatocellular carcinoma. Ann. Surg. Oncol..

[41] Wakabayashi T., Cacciaguerra A.B., Abe Y., Bona E.D., Nicolini D., Mocchegiani F., Kabeshima Y., Vivarelli M., Wakabayashi G., Kitagawa Y. (2022). Indocyanine Green Fluorescence Navigation in Liver Surgery: A Systematic Review on Dose and Timing of Administration. Ann. Surg..

[42] van der Vorst J.R., Schaafsma B.E., Hutteman M., Verbeek F.P.R., Liefers G.J., Hartgrink H.H., Smit V.T.H.B.M., Lowik C.W.G.M., van de Velde C.J.H., Frangioni J.V. (2013). Near-infrared fluorescence-guided resection of colorectal liver metastases. Cancer.

[43] Patel I., Bartlett D., Dasari B.V., Chatzizacharias N., Isaac J., Marudanayagam R., Mirza D.F., Roberts J.K., Sutcliffe R.P. (2022). Detection of Colorectal Liver Metastases Using Near-Infrared Fluorescence Imaging During Hepatectomy: Prospective Single Centre UK Study. J. Gastrointest. Cancer.

[44] Ishizawa T., Zuker N.B., Kokudo N., Gayet B. (2012). Positive and negative staining of hepatic segments by use of fluorescent imaging techniques during laparoscopic hepatectomy. Arch Surg..

[45] Newton A.D., Predina J.D., Shin M.H., Frenzel-Sulyok L.G., Vollmer C.M., Drebin J.A., Singhal S., Lee M.K.T. (2019). Intraoperative Near-infrared Imaging Can Identify Neoplasms and Aid in Real-time Margin Assessment During Pancreatic Resection. Ann. Surg..

[46] Hutteman M., van der Vorst J.R., Mieog J.S., Bonsing B.A., Hartgrink H.H., Kuppen P.J., Lowik C.W., Frangioni J.V., van de Velde C.J., Vahrmeijer A.L. (2011). Near-infrared fluorescence imaging in patients undergoing pancreaticoduodenectomy. Eur. Surg. Res..

[47] Lohman R.F., Ozturk C.N., Ozturk C., Jayaprakash V., Djohan R. (2015). An Analysis of Current Techniques Used for Intraoperative Flap Evaluation. Ann. Plast. Surg..

[48] Shirata C., Kawaguchi Y., Kobayashi K., Kobayashi Y., Arita J., Akamatsu N., Kaneko J., Sakamoto Y., Kokudo N., Hasegawa K. (2018). Usefulness of indocyanine green-fluorescence imaging for real-time visualization of pancreas neuroendocrine tumor and cystic neoplasm. J. Surg. Oncol..

[49] Paiella S., De Pastena M., Landoni L., Esposito A., Casetti L., Miotto M., Ramera M., Salvia R., Secchettin E., Bonamini D. (2017). Is there a role for near-infrared technology in laparoscopic resection of pancreatic neuroendocrine tumors? Results of the COLPAN “colour-and-resect the pancreas” study. Surg. Endosc..

[50] Rompianesi G., Montalti R., Giglio M.C., Ceresa C.D.L., Nasto R.A., De Simone G., Troisi R.I. (2022). Systematic review, meta-analysis and single-centre experience of the diagnostic accuracy of intraoperative near-infrared indocyanine green-fluorescence in detecting pancreatic tumours. HPB.

[51] Liberale G., Vankerckhove S., Caldon M.G., Ahmed B., Moreau M., Nakadi I.E., Larsimont D., Donckier V., Bourgeois P., Group R. (2016). Fluorescence Imaging After Indocyanine Green Injection for Detection of Peritoneal Metastases in Patients Undergoing Cytoreductive Surgery for Peritoneal Carcinomatosis from Colorectal Cancer: A Pilot Study. Ann. Surg..

[52] Leonhardt C.S., Niesen W., Kalkum E., Klotz R., Hank T., Buchler M.W., Strobel O., Probst P. (2022). Prognostic relevance of the revised R status definition in pancreatic cancer: Meta-analysis. BJS Open.

[53] Verbeke C.S., Menon K.V. (2009). Redefining resection margin status in pancreatic cancer. HPB.

[54] Strobel O., Hank T., Hinz U., Bergmann F., Schneider L., Springfeld C., Jager D., Schirmacher P., Hackert T., Buchler M.W. (2017). Pancreatic Cancer Surgery: The New R-status Counts. Ann. Surg..

[55] Hu Z., Fang C., Li B., Zhang Z., Cao C., Cai M., Su S., Sun X., Shi X., Li C. (2020). First-in-human liver-tumour surgery guided by multispectral fluorescence imaging in the visible and near-infrared-I/II windows. Nat. Biomed. Eng..

[56] Lauwerends L.J., van Driel P., Baatenburg de Jong R.J., Hardillo J.A.U., Koljenovic S., Puppels G., Mezzanotte L., Lowik C., Rosenthal E.L., Vahrmeijer A.L. (2021). Real-time fluorescence imaging in intraoperative decision making for cancer surgery. Lancet Oncol..

[57] Hoogstins C.E., Boogerd L.S., Sibinga Mulder B.G., Mieog J.S.D., Swijnenburg R.J., van de Velde C.J., Farina Sarasqueta A., Bonsing B.A., Framery B., Pèlegrin A. (2018). Image-Guided Surgery in Patients with Pancreatic Cancer: First Results of a Clinical Trial Using SGM-101, a Novel Carcinoembryonic Antigen-Targeting, Near-Infrared Fluorescent Agent. Ann. Surg. Oncol..

[58] McNally L.R., Mezera M., Morgan D.E., Frederick P.J., Yang E.S., Eltoum I.E., Grizzle W.E. (2016). Current and Emerging Clinical Applications of Multispectral Optoacoustic Tomography (MSOT) in Oncology. Clin. Cancer Res..

[59] Attia A.B.E., Balasundaram G., Moothanchery M., Dinish U.S., Bi R., Ntziachristos V., Olivo M. (2019). A review of clinical photoacoustic imaging: Current and future trends. Photoacoustics.

[60] Wang L.V., Hu S. (2012). Photoacoustic tomography: In vivo imaging from organelles to organs. Science.

[61] Hudson S.V., Huang J.S., Yin W., Albeituni S., Rush J., Khanal A., Yan J., Ceresa B.P., Frieboes H.B., McNally L.R. (2014). Targeted noninvasive imaging of EGFR-expressing orthotopic pancreatic cancer using multispectral optoacoustic tomography. Cancer Res..

[62] Chen Z., Dean-Ben X.L., Gottschalk S., Razansky D. (2018). Performance of optoacoustic and fluorescence imaging in detecting deep-seated fluorescent agents. Biomed. Opt. Express.

[63] Bhutiani N., Kimbrough C.W., Burton N.C., Morscher S., Egger M., McMasters K., Woloszynska-Read A., El-Baz A., McNally L.R. (2017). Detection of microspheres in vivo using multispectral optoacoustic tomography. Biotech. Histochem..

[64] MacCuaig W.M., Jones M.A., Abeyakoon O., McNally L.R. (2020). Development of Multispectral Optoacoustic Tomography as a Clinically Translatable Modality for Cancer Imaging. Radiol. Imaging Cancer.

[65] Bagley A.F., Ludmir E.B., Maitra A., Minsky B.D., Li Smith G., Das P., Koong A.C., Holliday E.B., Taniguchi C.M., Katz M.H.G. (2022). NBTXR3, a first-in-class radioenhancer for pancreatic ductal adenocarcinoma: Report of first patient experience. Clin. Transl. Radiat. Oncol..

[66] Dennahy I.S., Han Z., MacCuaig W.M., Chalfant H.M., Condacse A., Hagood J.M., Claros-Sorto J.C., Razaq W., Holter-Chakrabarty J., Squires R. (2022). Nanotheranostics for Image-Guided Cancer Treatment. Pharmaceutics.

[67] Herzog E., Taruttis A., Beziere N., Lutich A.A., Razansky D., Ntziachristos V. (2012). Optical imaging of cancer heterogeneity with multispectral optoacoustic tomography. Radiology.

[68] Gargiulo S., Albanese S., Mancini M. (2019). State-of-the-Art Preclinical Photoacoustic Imaging in Oncology: Recent Advances in Cancer Theranostics. Contrast Media Mol. Imaging.

[69] Ntziachristos V., Pleitez M.A., Aime S., Brindle K.M. (2019). Emerging Technologies to Image Tissue Metabolism. Cell Metab..

[70] Laramie M.D., Fouts B.L., MacCuaig W.M., Buabeng E., Jones M.A., Mukherjee P., Behkam B., McNally L.R., Henary M. (2021). Improved pentamethine cyanine nanosensors for optoacoustic imaging of pancreatic cancer. Sci. Rep..

[71] MacCuaig W.M., Fouts B.L., McNally M.W., Grizzle W.E., Chuong P., Samykutty A., Mukherjee P., Li M., Jasinski J.B., Behkam B. (2021). Active Targeting Significantly Outperforms Nanoparticle Size in Facilitating Tumor-Specific Uptake in Orthotopic Pancreatic Cancer. ACS Appl. Mater. Interfaces.

[72] Cao R., Kilroy J.P., Ning B., Wang T., Hossack J.A., Hu S. (2015). Multispectral photoacoustic microscopy based on an optical-acoustic objective. Photoacoustics.

[73] Samykutty A., Thomas K.N., McNally M., Hagood J., Chiba A., Thomas A., McWilliams L., Behkam B., Zhan Y., Council-Troche M. (2023). Simultaneous Detection of Multiple Tumor-targeted Gold Nanoparticles in HER2-Positive Breast Tumors Using Optoacoustic Imaging. Radiol. Imaging Cancer.

[74] Chae H.D., Lee J.Y., Jang J.Y., Chang J.H., Kang J., Kang M.J., Han J.K. (2017). Photoacoustic Imaging for Differential Diagnosis of Benign Polyps versus Malignant Polyps of the Gallbladder: A Preliminary Study. Korean J. Radiol..

[75] Kimbrough C.W., Hudson S., Khanal A., Egger M.E., McNally L.R. (2015). Orthotopic pancreatic tumors detected by optoacoustic tomography using Syndecan-1. J. Surg. Res..

[76] Bhutiani N., Grizzle W.E., Galandiuk S., Otali D., Dryden G.W., Egilmez N.K., McNally L.R. (2017). Noninvasive Imaging of Colitis Using Multispectral Optoacoustic Tomography. J. Nucl. Med..

[77] Harold K.M., MacCuaig W.M., Holter-Charkabarty J., Williams K., Hill K., Arreola A.X., Sekhri M., Carter S., Gomez-Gutierrez J., Salem G. (2022). Advances in Imaging of Inflammation, Fibrosis, and Cancer in the Gastrointestinal Tract. Int. J. Mol. Sci..

[78] Ogawa M., Kosaka N., Choyke P.L., Kobayashi H. (2009). In vivo molecular imaging of cancer with a quenching near-infrared fluorescent probe using conjugates of monoclonal antibodies and indocyanine green. Cancer Res..

[79] Chalfant H., Bonds M., Scott K., Condacse A., Dennahy I.S., Martin W.T., Little C., Edil B.H., McNally L.R., Jain A. (2023). Innovative Imaging Techniques Used to Evaluate Borderline-Resectable Pancreatic Adenocarcinoma. J. Surg. Res..

[80] Han Z., MacCuaig W.M., Gurcan M.N., Claros-Sorto J., Garwe T., Henson C., Holter-Chakrabarty J., Hannafon B., Chandra V., Wellberg E. (2023). Dynamic 2-deoxy-D-glucose-enhanced multispectral optoacoustic tomography for assessing metabolism and vascular hemodynamics of breast cancer. Photoacoustics.

[81] Bohndiek S.E., Sasportas L.S., Machtaler S., Jokerst J.V., Hori S., Gambhir S.S. (2015). Photoacoustic Tomography Detects Early Vessel Regression and Normalization During Ovarian Tumor Response to the Antiangiogenic Therapy Trebananib. J. Nucl. Med..

[82] Tummers W.S., Miller S.E., Teraphongphom N.T., Gomez A., Steinberg I., Huland D.M., Hong S., Kothapalli S.R., Hasan A., Ertsey R. (2018). Intraoperative Pancreatic Cancer Detection using Tumor-Specific Multimodality Molecular Imaging. Ann. Surg. Oncol..

[83] Napp J., Stammes M.A., Claussen J., Prevoo H., Sier C.F.M., Hoeben F.J.M., Robillard M.S., Vahrmeijer A.L., Devling T., Chan A.B. (2018). Fluorescence- and multispectral optoacoustic imaging for an optimized detection of deeply located tumors in an orthotopic mouse model of pancreatic carcinoma. Int. J. Cancer.

[84] Nishimura M., Murayama Y., Harada K., Kamada Y., Morimura R., Ikoma H., Ichikawa D., Fujiwara H., Okamoto K., Otsuji E. (2016). Photodynamic Diagnosis of Hepatocellular Carcinoma Using 5-Aminolevulinic Acid. Anticancer Res..

[85] Inoue Y., Tanaka R., Komeda K., Hirokawa F., Hayashi M., Uchiyama K. (2014). Fluorescence detection of malignant liver tumors using 5-aminolevulinic acid-mediated photodynamic diagnosis: Principles, technique, and clinical experience. World J. Surg..

[86] Fujiwara H., Takahara N., Tateishi K., Tanaka M., Kanai S., Kato H., Nakatsuka T., Yamamoto K., Kogure H., Arita J. (2020). 5-Aminolevulinic acid-mediated photodynamic activity in patient-derived cholangiocarcinoma organoids. Surg. Oncol..

[87] Saito T., Ebihara Y., Li L., Shirosaki T., Iijima H., Tanaka K., Nakanishi Y., Asano T., Noji T., Kurashima Y. (2021). A novel laparoscopic near-infrared fluorescence spectrum system for photodynamic diagnosis of peritoneal dissemination in pancreatic cancer. Photodiagn. Photodyn. Ther..

[88] Abrahamse H., Hamblin M.R. (2016). New photosensitizers for photodynamic therapy. Biochem. J..

[89] Alsaab H.O., Alghamdi M.S., Alotaibi A.S., Alzhrani R., Alwuthaynani F., Althobaiti Y.S., Almalki A.H., Sau S., Iyer A.K. (2020). Progress in Clinical Trials of Photodynamic Therapy for Solid Tumors and the Role of Nanomedicine. Cancers.

[90] Hanada Y., Pereira S.P., Pogue B., Maytin E.V., Hasan T., Linn B., Mangels-Dick T., Wang K.K. (2021). EUS-guided verteporfin photodynamic therapy for pancreatic cancer. Gastrointest. Endosc..

[91] Fang C., Zhang P., Qi X. (2019). Digital and intelligent liver surgery in the new era: Prospects and dilemmas. EBioMedicine.

[92] Felli E., Boleslawski E., Sommacale D., Scatton O., Brustia R., Schwarz L., Cherqui D., Zacharias T., Laurent A., Mabrut J.Y. (2023). Paradigm shift: Should preoperative 3D reconstruction models become mandatory before hepatectomy for hepatocellular carcinoma (HCC)? Results of a multicenter prospective trial. HPB.

[93] Saito Y., Sugimoto M., Imura S., Morine Y., Ikemoto T., Iwahashi S., Yamada S., Shimada M. (2020). Intraoperative 3D Hologram Support with Mixed Reality Techniques in Liver Surgery. Ann. Surg..

[94] Liu J.P., Lerut J., Yang Z., Li Z.K., Zheng S.S. (2022). Three-dimensional modeling in complex liver surgery and liver transplantation. Hepatobiliary Pancreat. Dis. Int..

[95] Maffione A.M., Rubello D., Caroli P., Colletti P.M., Matteucci F. (2020). Is It Time to Introduce PET/CT in Colon Cancer Guidelines?. Clin. Nucl. Med..

[96] Sacks A., Peller P.J., Surasi D.S., Chatburn L., Mercier G., Subramaniam R.M. (2011). Value of PET/CT in the management of primary hepatobiliary tumors, part 2. AJR Am. J. Roentgenol..

[97] Tsang V.T.C., Li X., Wong T.T.W. (2020). A Review of Endogenous and Exogenous Contrast Agents Used in Photoacoustic Tomography with Different Sensing Configurations. Sensors.

[98] Zhao Z., Swartchick C.B., Chan J. (2022). Targeted contrast agents and activatable probes for photoacoustic imaging of cancer. Chem. Soc. Rev..

[99] Kaplon H., Crescioli S., Chenoweth A., Visweswaraiah J., Reichert J.M. (2023). Antibodies to watch in 2023. MAbs.

[100] Bever K.M., Sugar E.A., Bigelow E., Sharma R., Laheru D., Wolfgang C.L., Jaffee E.M., Anders R.A., De Jesus-Acosta A., Zheng L. (2015). The prognostic value of stroma in pancreatic cancer in patients receiving adjuvant therapy. HPB.

[101] England C.G., Hernandez R., Eddine S.B., Cai W. (2016). Molecular Imaging of Pancreatic Cancer with Antibodies. Mol. Pharm..

[102] Catenacci D.V. (2015). Next-generation clinical trials: Novel strategies to address the challenge of tumor molecular heterogeneity. Mol. Oncol..

[103] Mulder B.G.S., Koller M., Duiker E.W., Sarasqueta A.F., Burggraaf J., Meijer V.E., Vahrmeijer A.L., Hoogwater F.J.H., Bonsing B.A., van Dam G.M. (2023). Intraoperative Molecular Fluorescence Imaging of Pancreatic Cancer by Targeting Vascular Endothelial Growth Factor: A Multicenter Feasibility Dose-Escalation Study. J. Nucl. Med..

[104] de Gooyer J.M., Elekonawo F.M., Bos D.L., van der Post R.S., Pèlegrin A., Framery B., Cailler F., Vahrmeijer A.L., de Wilt J.H., Rijpkema M. (2020). Multimodal CEA-targeted image-guided colorectal cancer surgery using 111In-labeled SGM-101. Clin. Cancer Res..

[105] Bannas P., Lenz A., Kunick V., Well L., Fumey W., Rissiek B., Haag F., Schmid J., Schutze K., Eichhoff A. (2015). Molecular imaging of tumors with nanobodies and antibodies: Timing and dosage are crucial factors for improved in vivo detection. Contrast Media Mol. Imaging.

[106] Baart V.M., van Manen L., Bhairosingh S.S., Vuijk F.A., Iamele L., de Jonge H., Scotti C., Resnati M., Cordfunke R.A., Kuppen P.J.K. (2021). Side-by-Side Comparison of uPAR-Targeting Optical Imaging Antibodies and Antibody Fragments for Fluorescence-Guided Surgery of Solid Tumors. Mol. Imaging Biol..

[107] Debie P., Devoogdt N., Hernot S. (2019). Targeted Nanobody-Based Molecular Tracers for Nuclear Imaging and Image-Guided Surgery. Antibodies.

[108] Zettlitz K.A., Tsai W.K., Knowles S.M., Kobayashi N., Donahue T.R., Reiter R.E., Wu A.M. (2018). Dual-Modality Immuno-PET and Near-Infrared Fluorescence Imaging of Pancreatic Cancer Using an Anti-Prostate Stem Cell Antigen Cys-Diabody. J. Nucl. Med..

[109] Kimbrough C.W., Khanal A., Zeiderman M., Khanal B.R., Burton N.C., McMasters K.M., Vickers S.M., Grizzle W.E., McNally L.R. (2015). Targeting Acidity in Pancreatic Adenocarcinoma: Multispectral Optoacoustic Tomography Detects pH-Low Insertion Peptide Probes In Vivo. Clin. Cancer Res..

[110] Jing R., Zhou X., Zhao J., Wei Y., Zuo B., You A., Rao Q., Gao X., Yang R., Chen L. (2018). Fluorescent peptide highlights micronodules in murine hepatocellular carcinoma models and humans in vitro. Hepatology.

[111] Shi J., Wang F., Liu S. (2016). Radiolabeled cyclic RGD peptides as radiotracers for tumor imaging. Biophys. Rep..

[112] Zhang G.Q., Zhong L.P., Yang N., Zhao Y.X. (2019). Screening of aptamers and their potential application in targeted diagnosis and therapy of liver cancer. World J. Gastroenterol..

[113] Li F.Q., Zhang S.X., An L.X., Gu Y.Q. (2011). In vivo molecular targeting effects of anti-Sp17- ICG-Der-02 on hepatocellular carcinoma evaluated by an optical imaging system. J. Exp. Clin. Cancer Res..

[114] Fu R., Carroll L., Yahioglu G., Aboagye E.O., Miller P.W. (2018). Antibody Fragment and Affibody ImmunoPET Imaging Agents: Radiolabelling Strategies and Applications. ChemMedChem.

[115] Gurka M.K., Pender D., Chuong P., Fouts B.L., Sobelov A., McNally M.W., Mezera M., Woo S.Y., McNally L.R. (2016). Identification of pancreatic tumors in vivo with ligand-targeted, pH responsive mesoporous silica nanoparticles by multispectral optoacoustic tomography. J. Control. Release.

[116] Ingels A., Leguerney I., Cournede P.H., Irani J., Ferlicot S., Sebrie C., Benatsou B., Jourdain L., Pitre-Champagnat S., Patard J.J. (2020). Ultrasound Molecular Imaging of Renal Cell Carcinoma: VEGFR targeted therapy monitored with VEGFR1 and FSHR targeted microbubbles. Sci. Rep..

[117] Nishino H., Turner M.A., Amirfakhri S., Lwin T.M., Hosseini M., Singer B.B., Hoffman R.M., Bouvet M. (2023). Proof of Principle of Combining Fluorescence-Guided Surgery with Photoimmunotherapy to Improve the Outcome of Pancreatic Cancer Therapy in an Orthotopic Mouse Model. Ann. Surg. Oncol..

[118] Park J.Y., Hiroshima Y., Lee J.Y., Maawy A.A., Hoffman R.M., Bouvet M. (2015). MUC1 selectively targets human pancreatic cancer in orthotopic nude mouse models. PLoS ONE.

[119] Handgraaf H.J.M., Boonstra M.C., Prevoo H., Kuil J., Bordo M.W., Boogerd L.S.F., Sibinga Mulder B.G., Sier C.F.M., Vinkenburg-van Slooten M.L., Valentijn A. (2017). Real-time near-infrared fluorescence imaging using cRGD-ZW800-1 for intraoperative visualization of multiple cancer types. Oncotarget.

[120] Boonstra M.C., Tolner B., Schaafsma B.E., Boogerd L.S., Prevoo H.A., Bhavsar G., Kuppen P.J., Sier C.F., Bonsing B.A., Frangioni J.V. (2015). Preclinical evaluation of a novel CEA-targeting near-infrared fluorescent tracer delineating colorectal and pancreatic tumors. Int. J. Cancer.

[121] Deng H., Shang W.T., Lu G.H., Guo P.Y., Ai T., Fang C.H., Tian J. (2019). Targeted and Multifunctional Technology for Identification between Hepatocellular Carcinoma and Liver Cirrhosis. ACS Appl. Mater. Interfaces.

[122] Yan H., Gao X., Zhang Y., Chang W., Li J., Li X., Du Q., Li C. (2018). Imaging Tiny Hepatic Tumor Xenografts via Endoglin-Targeted Paramagnetic/Optical Nanoprobe. ACS Appl. Mater. Interfaces.

[123] Zeng Z., Chen J., Luo S., Dong J., Hu H., Yang Z., Feng X., Liu Y., Liu B., Pan G. (2020). Targeting and imaging colorectal cancer by activatable cell-penetrating peptides. Am. J. Transl. Res..

[124] Jin Y., Wang K., Tian J. (2018). Preoperative Examination and Intraoperative Identification of Hepatocellular Carcinoma Using a Targeted Bimodal Imaging Probe. Bioconjug. Chem..

[125] Hernandez R., Sun H., England C.G., Valdovinos H.F., Ehlerding E.B., Barnhart T.E., Yang Y., Cai W. (2016). CD146-targeted immunoPET and NIRF Imaging of Hepatocellular Carcinoma with a Dual-Labeled Monoclonal Antibody. Theranostics.

[126] Hollandsworth H.M., Nishino H., Turner M., Amirfakhri S., Filemoni F., Hoffman R.M., Yazaki P.J., Bouvet M. (2020). Humanized Fluorescent Tumor-associated Glycoprotein-72 Antibody Selectively Labels Colon-cancer Liver Metastases in Orthotopic Mouse Models. In Vivo.

[127] Zeng C., Shang W., Wang K., Chi C., Jia X., Fang C., Yang D., Ye J., Fang C., Tian J. (2016). Intraoperative Identification of Liver Cancer Microfoci Using a Targeted Near-Infrared Fluorescent Probe for Imaging-Guided Surgery. Sci. Rep..

[128] Tang C., Du Y., Liang Q., Cheng Z., Tian J. (2020). Development of a Novel Histone Deacetylase-Targeted Near-Infrared Probe for Hepatocellular Carcinoma Imaging and Fluorescence Image-Guided Surgery. Mol. Imaging Biol..

[129] Hiroshima Y., Lwin T.M., Murakami T., Mawy A.A., Kuniya T., Chishima T., Endo I., Clary B.M., Hoffman R.M., Bouvet M. (2016). Effective fluorescence-guided surgery of liver metastasis using a fluorescent anti-CEA antibody. J. Surg. Oncol..

[130] Zhao M., Dong L., Liu Z., Yang S., Wu W., Lin J. (2018). In vivo fluorescence imaging of hepatocellular carcinoma using a novel GPC3-specific aptamer probe. Quant. Imaging Med. Surg..

[131] Qi S., Liu G., Chen J., Cao P., Lei X., Ding C., Chen G., Zhang Y., Wang L. (2022). Targeted Multifunctional Nanoplatform for Imaging-Guided Precision Diagnosis and Photothermal/Photodynamic Therapy of Orthotopic Hepatocellular Carcinoma. Int. J. Nanomed..

[132] Chen Y., Lu J., Yang J., Hao K., Li M. (2020). Investigation of Alpha-Fetoprotein Antibody Modified Fluorescent Magnetic Probe on HepG(2) Cell and Cancer Model Mouse. J. Nanosci. Nanotechnol..

[133] Park J.Y., Murakami T., Lee J.Y., Zhang Y., Hoffman R.M., Bouvet M. (2016). Fluorescent-Antibody Targeting of Insulin-Like Growth Factor-1 Receptor Visualizes Metastatic Human Colon Cancer in Orthotopic Mouse Models. PLoS ONE.

[134] Wu L.Y., Ishigaki Y., Hu Y.X., Sugimoto K., Zeng W.H., Harimoto T., Sun Y.D., He J., Suzuki T., Jiang X.Q. (2020). H_2_S-activatable near-infrared afterglow luminescent probes for sensitive molecular imaging in vivo. Nat. Commun..

[135] Brachi G., Bussolino F., Ciardelli G., Mattu C. (2019). Nanomedicine for Imaging and Therapy of Pancreatic Adenocarcinoma. Front. Bioeng. Biotechnol..

[136] Valderrama-Trevino A.I., Barrera-Mera B., Ceballos-Villalva J.C., Montalvo-Jave E.E. (2017). Hepatic Metastasis from Colorectal Cancer. Euroasian J. Hepatogastroenterol..

[137] Chan A., Zhang W.Y., Chok K., Dai J., Ji R., Kwan C., Man N., Poon R., Lo C.M. (2021). ALPPS Versus Portal Vein Embolization for Hepatitis-related Hepatocellular Carcinoma: A Changing Paradigm in Modulation of Future Liver Remnant Before Major Hepatectomy. Ann. Surg..

[138] Joliat G.R., Kobayashi K., Hasegawa K., Thomson J.E., Padbury R., Scott M., Brustia R., Scatton O., Tran Cao H.S., Vauthey J.N. (2023). Guidelines for Perioperative Care for Liver Surgery: Enhanced Recovery After Surgery (ERAS) Society Recommendations 2022. World J. Surg..

[139] De Simoni O., Scarpa M., Tonello M., Pilati P., Tolin F., Spolverato Y., Gruppo M. (2020). Oligometastatic Pancreatic Cancer to the Liver in the Era of Neoadjuvant Chemotherapy: Which Role for Conversion Surgery? A Systematic Review and Meta-Analysis. Cancers.

[140] Martin J., Petrillo A., Smyth E.C., Shaida N., Khwaja S., Cheow H.K., Duckworth A., Heister P., Praseedom R., Jah A. (2020). Colorectal liver metastases: Current management and future perspectives. World J. Clin. Oncol..

